# Selenium against lead-induced apoptosis in chicken nervous tissues via mitochondrial pathway

**DOI:** 10.18632/oncotarget.22553

**Published:** 2017-11-20

**Authors:** Yihao Zhu, Xiaoyan Jiao, Yang An, Shu Li, Xiaohua Teng

**Affiliations:** ^1^ College of Animal Science and Technology, Northeast Agricultural University, Harbin 150030, People's Republic of China; ^2^ College of Veterinary Medicine, Northeast Agricultural University, Harbin 150030, People's Republic of China

**Keywords:** lead, selenium, chicken nervous tissue, apoptosis, mitochondrial pathway

## Abstract

To investigate alleviative effect of selenium (Se) on lead (Pb)-induced apoptosis in chicken nervous tissues, 7-day-old chickens were randomly divided into four groups. The control group was fed a standard diet and drinking water. In the Pb and Se/Pb groups, (CH_3_OO)_2_Pb was dissolved in drinking water. In the Se and Se/Pb groups, Na_2_SeO_3_ was put into the standard diet. Embryonic neurocytes were divided into the control, Se (containing Na_2_SeO_3_), Pb (containing (CH_3_COO)_2_Pb), and Se/Pb (containing Na_2_SeO_3_ and (CH_3_COO)_2_Pb) groups. The following contents were performed: Morphologic observation for 90 days in brain tissues and for 12, 24, 36, and 48 hours in embryonic neurocytes; and antioxidant indexes, messenger RNA (mRNA) expression of twenty-five selenoproteins, and mRNA and protein expression of five apoptosis-related genes for 30, 60, and 90 days in brain tissues and for 12, 24, 36, and 48 hours in embryonic neurocytes. The results indicated that Se alleviated Pb-caused morphological changes; the decrease of superoxide dismutase, glutathione peroxidase (GPx), GPx1, GPx2, GPx3, GPx4, thioredoxin reductases (Txnrd)1, Txnrd2, Txnrd3, iodothyronine deiodinases (Dio)1, Dio2, Dio3, selenoprotein (Sel)T, SelK, SelS, SelH, SelM, SelU, SelI, SelO, Selpb, selenoprotein (Sep)n1, Sepp1, Sepx1, Sepw1, 15-kDa selenoprotein, and selenophosphate synthetases 2, and B-cell lymphoma-2 (Bcl-2); the increase of malondialdehyde, p53, Bcl-2 associated X protein, cytochrome c, and Caspase-3. Pb had time-dependent effects on GPx4, SelM, and malondialdehyde in the brain tissues; and on SelU in the embryonic neurocytes. Our data demonstrated that Se alleviated Pb-induced apoptosis in the chicken nervous tissues via mitochondrial pathway.

## INTRODUCTION

Lead (Pb) is a toxic heavy metal and can cause bird and human poisoning through contaminated soil and vegetation. Pb caused topsoil and vegetation pollution near Pb battery manufacture factory in Ibadan, Nigeria [1]. Pb poisoning was diagnosed in 8% of birds in mallards and coots from two hunting activity areas in Poland [2]. Children and adolescents had neurocognitive deficit in a local ceramic glazing cottage industry in Ecuadorian villages with Pb exposure [3]. Occupational exposure to Pb caused cognitive deficit in Pittsburgh battery workers [4]. Pb caused behavioral changes of wild birds in Belgium hunting areas [5]. Excess Pb caused human and animal neurotoxicity, including behavioral deficit in herring gull brains [6], hippocampus damage and apoptosis in rat adrenal medulla pheochromocytoma cells (PC 12) [7], oxidative stress and apoptosis in human neuroblastoma cells [8], and apoptosis in rat brains [9]. Cadmium (Cd) disrupted mitochondrial membrane integrity and triggered release of mitochondrial proteins cytochrome c (Cyt c) into cytosol, then Cyt c activated Caspase-3, finally apoptosis occurred in PC 12 [10]. Pb induced apoptosis via mitochondrial pathway in rat proximal tubular cells [11]. B-cell lymphoma-2 (Bcl-2), p53, Bcl-2 associated X protein (Bax), Cyt c, and Caspase-3 involved in Pb-induced apoptosis in rat brains [9]. Therefore, we wanted to study neurotoxicity via inducing apoptosis in birds.

Selenium (Se) is an essential trace element in organisms [12]. Se alleviated Pb-caused the decrease of neural cell adhesion molecules in Wistar rat blood and hippocampus [13], and Cd-induced oxidative stress and apoptosis in mouse kidneys [14]. Se exerts its biological activity through incorporating into selenoproteins in form of selenocysteine [15]. Glutathione peroxidases (GPx)1, GPx2, GPx3, GPx4, thioredoxin reductases (Txnrd)1, Txnrd2, Txnrd3, iodothyronine deiodinases (Dio)1, Dio2, Dio3, selenoprotein (Sel)T, SelK, SelS, SelH, SelM, SelU, SelI, SelO, Selpb, selenoprotein (Sep)n1, Sepp1, Sepx1, Sepw1, 15-kDa selenoprotein (Sep15), and selenophosphate synthetases 2 (SPS2) are selenoproteins in animals and humans [16]. Se can alleviate Cd-caused the decrease of GPx4 expression in rat testes [17] and the decrease of SelT, SelK, and SelS messenger RNA (mRNA) expression in chicken splenic lymphocytes [18]. SelT, SelK, and SelS may be related to protection of Se against Cd toxicity in chicken splenic lymphocytes [18]. Se is a cofactor of GPx, which is an antioxidant enzyme [18]. SelP and GPx participated in protection of human astrocytes from tert-butylhydroperoxide-induced oxidative damage [19]. Antioxidant of Se may be a mechanism of protective effect against Pb toxicity [20]. It was found that Se can antagonize Cd-induced apoptosis via mitochondrial pathway in chicken splenic lymphocytes [21] and in mouse kidneys [14].

Studies about alleviative effect of Se on Pb poisoning in chickens were focused on *in vivo*, such as cartilages about mRNA expression of GPx1, GPx2, GPx3, GPx4, Txnrd1, Txnrd2, Txnrd3, Dio1, Dio2, Dio3, SelT, SelK, SelS, SelH, SelM, SelU, SelI, SelO, Selpb, Sepp1, Sepx1, Sep15, and SPS2 [22]; kidneys about mitochondrial dynamics and protein expression of Bcl-2, p53, Bax, Cyt c, and Caspase-3 [23]; bursa of Fabricius about oxidative stress indicators and cytokines mRNA expression [24]; and testes about mRNA and protein expression of inflammatory factors and heat shock proteins [25], mRNA expression of heat shock proteins and GPx1, GPx2, GPx3, GPx4, Txnrd1, Txnrd2, Txnrd3, Dio1, Dio2, Dio3, SelT, SelK, SelS, SelH, SelM, SelU, SelI, SelO, Selpb, Selpn1, Sepp1, Sepx1, Sepw1, Sep15, and SPS2 [26]. However, little is known about alleviative effect of Se on Pb-caused apoptosis in bird nervous tissues *in vivo* and *in vitro* via mitochondrial pathway. Therefore, in this study, we wanted to establish chicken and chicken embryonic neurocytes models of alleviative effect of Se on Pb poisoning *in vivo* and *in vitro*; observe morphological changes; examine superoxide dismutase (SOD) and GPx activities, malondialdehyde (MDA) content, mRNA expression of twenty-five selenoproteins (GPx1, GPx2, GPx3, GPx4, Txnrd1, Txnrd2, Txnrd3, Dio1, Dio2, Dio3, SelT, SelK, SelS, SelH, SelM, SelU, SelI, SelO, Selpb, Sepn1, Sepp1, Sepx1, Sepw1, Sep15, and SPS2), and mRNA and protein expression of five apoptosis-related genes (Bcl-2, p53, Bax, Cyt c, and Caspase-3); and investigate alleviative effect of Se on Pb-induced apoptosis in chicken nervous tissues via mitochondrial pathway.

## RESULTS

### Cell viability

**Figure 1:**
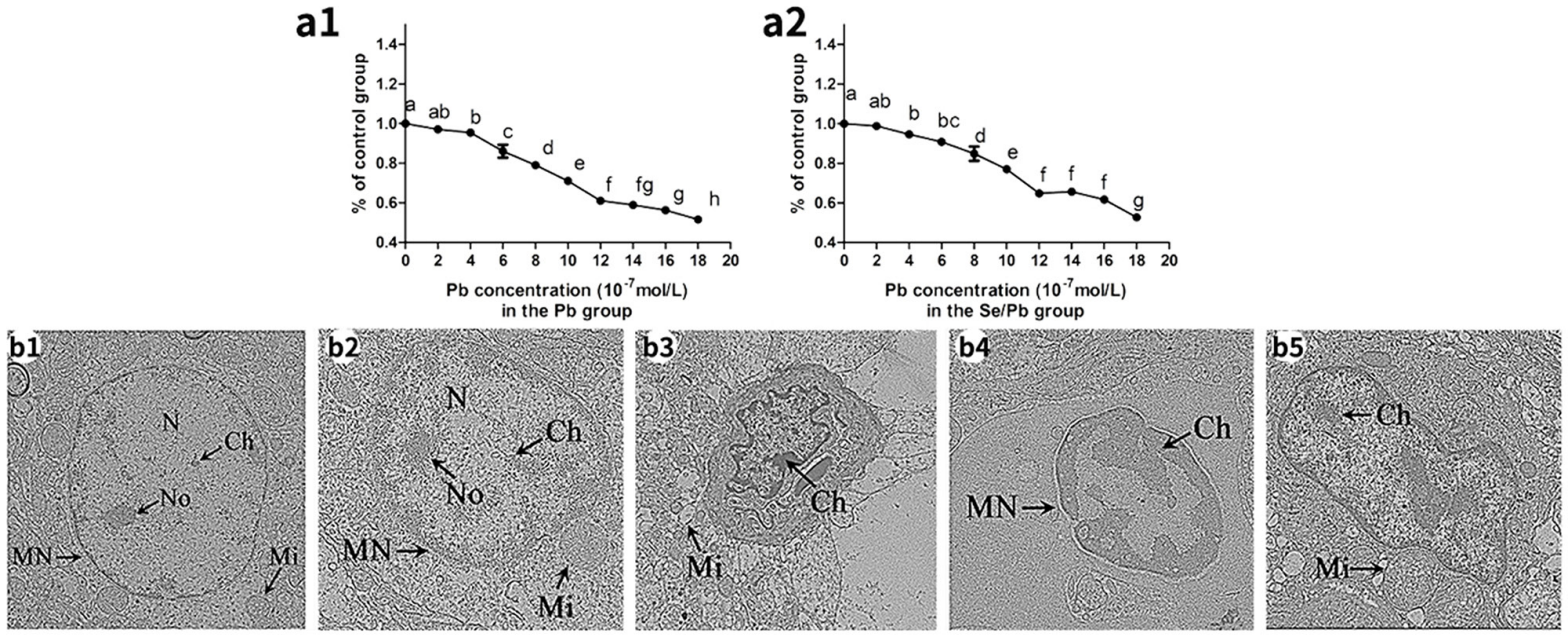
Cell viabilities of the chicken embryonic neurocytes for 48 hours and the ultrastructure of the chicken brain tissues on the 90^th^ day *Different lowercase letters* indicate significant differences (*P* < 0.05). **(a1)**: Cell viability in the Pb group; **(a2)**: cell viability in the Se/Pb group; **(b1)**: the control group; **(b2)**: the Se group; **(b3)**: the Pb group; **(b4)**: the Pb group; **(b5)**: the Se/Pb group. Magnification: b1 × 30,000; b2 × 25,000; b3 × 15,000; b4 × 25,000; b5 × 20,000.

As shown in Figure 1, 50% inhibitory concentration (IC50) of chicken embryonic neurocytes for 48 hours in the Pb (Figure 1a1) and Se/Pb groups (Figure 1a2) were measured. Cell viabilities in the Pb group were 97.07%, 95.46%, 86.06%, 79.00%, 71.03%, 61.03%, 58.91%, 56.24%, and 56.24%, respectively, with Pb concentrations of 2, 4, 6, 8, 10, 12, 14, 16, and 18 × 10^−7^ mol/L. Cell viabilities in the Se/Pb group were 98.82%, 94.54%, 90.80%, 84.86%, 77.02%, 64.81%, 65.66%, 61.66%, and 52.66%, respectively, with Se concentration of 10 × 10^−8^ mol/L and Pb concentrations of 2, 4, 6, 8, 10, 12, 14, 16, and 18 × 10^−7^ mol/L. Cell viabilities in the Pb and Se/Pb groups with 18 × 10^−7^ mol/L Pb were 56.24% and 52.66%, respectively. IC50 of Pb at 48 hours was 18 × 10^−7^ mol/L. Cell viabilities of chicken embryonic neurocytes in the Pb and Se/Pb groups with Pb concentration of 10 × 10^−7^ mol/L were 71.03% and 77.02%, respectively. Pb concentration of 10 × 10^−7^ mol/L was equivalent to 5/9 of IC50. Therefore, Se concentration of 10 × 10^−8^ mol/L and Pb concentration of 10 × 10^−7^ mol/L for 48 hours were selected in the experiment.

### Apoptosis observation in chicken brain tissues

Ultrastructure observation of chicken brain tissues on the 90^th^ day was shown in Figure 1. In the control (Figure 1b1) and Se (Figure 1b2) groups, cells were normal with complete nucleus (N) and nucleolus (No), intact mitochondria (Mi), and normal chromatin (Ch). In the Pb group, nucleus became shrunk (Figure 1b3), swollen mitochondria appeared (Figure 1b3), chromatin margination was clearly present (Figure 1b3 and 1b4), abnormal nuclear shape occurred (Figure 1b3 and 1b4), nucleolus disappeared (Figure 1b4), and nuclear membrane fusion appeared (Figure 1b4). In the Se/Pb group (Figure 1b5), degrees of abnormal nuclear shape, chromatin margination, and swollen mitochondria were lower than those in the Pb group.

### Apoptosis observation in chicken embryonic neurocytes

**Figure 2:**
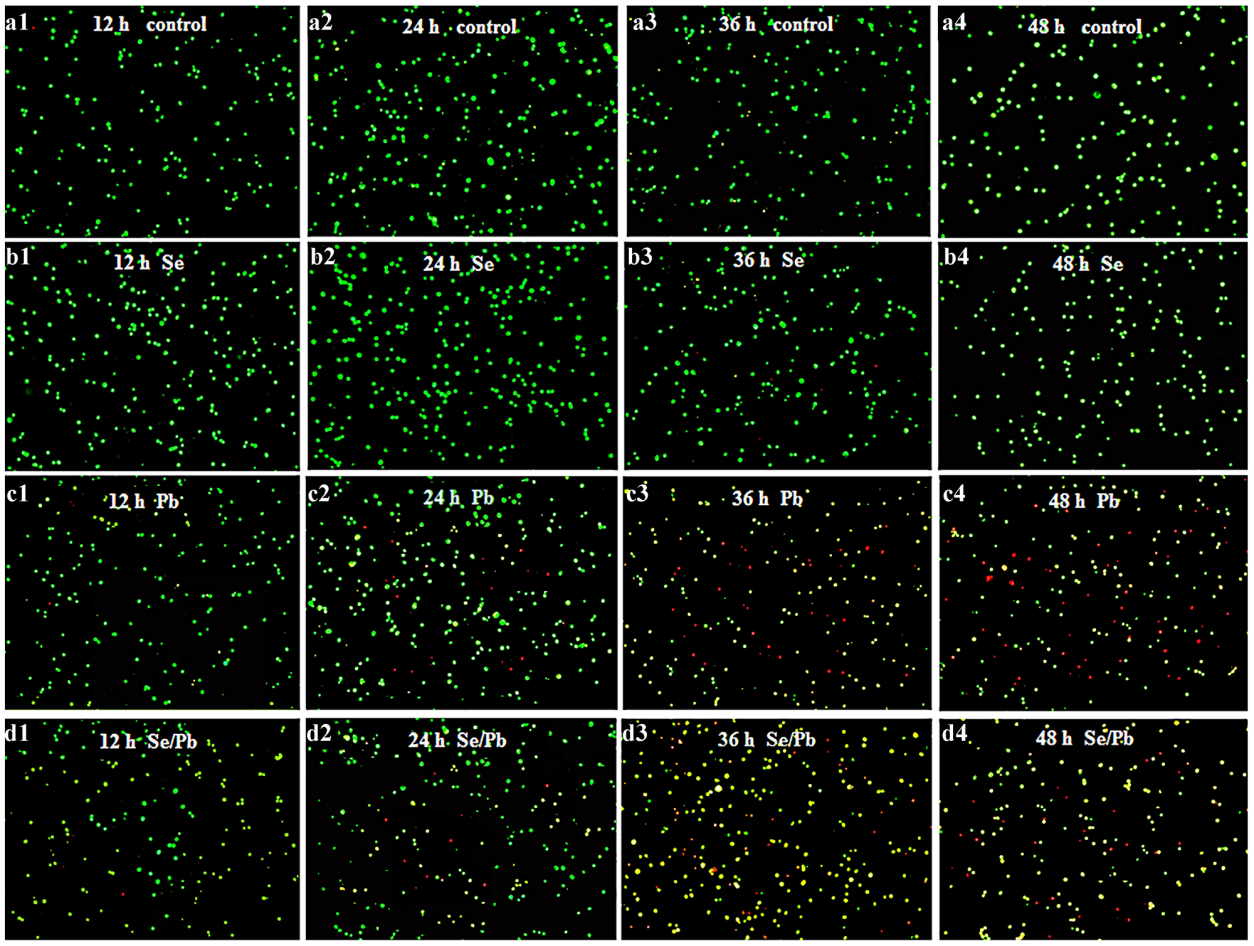
Apoptosis observation in the chicken embryonic neurocytes **(a)**: The control groups; **(b)**: the Se groups; **(c)**: the Pb groups; **(d)**: the Se/Pb groups; **(1)**: 12 hours; **(2)**: 24 hours; **(3)**: 36 hours: **(4)**: 48 hours.

In this experiment, apoptosis observation of chicken embryonic neurocytes was performed. As shown in Figure 2, in the control (Figure 2a1-2a4) and Se (Figure 2b1-2b4) groups at all the time points, cells were round and green with the same size. In the Pb group for 12 (Figure 2c1), 24 (Figure 2c2), 36 (Figure 2c3), and 48 hours (Figure 2c4), cells showed irregular shape. Chromatin condensation occurred and there were yellow and red cells. Degree of irregular shape and numbers of yellow and red cells increased with the increase of treatment time. In the Se/Pb group for 12 (Figure 2d1), 24 (Figure 2d2), 36 (Figure 2d3), and 48 hours (Figure 2d4), irregular degree of cells, the degree of chromatin condensation, and the numbers of yellow and red cells increased with the increase of treatment time. Irregular degree of cells and the degree of chromatin condensation in the Se/Pb group were lower than those in the Pb group at all the same time points. Numbers of yellow and red cells in the Se/Pb group were less than those in the Pb group at all the same time points.

### Relative mRNA expression of twenty-five selenoproteins in chicken brain tissues and embryonic neurocytes

Relative mRNA expression of twenty-five selenoproteins (GPx1, GPx2, GPx3, GPx4, Txnrd1, Txnrd2, Txnrd3, Dio1, Dio2, Dio3, SelT, SelK, SelS, SelH, SelM, SelU, SelI, SelO, Selpb, Sepn1, Sepp1, Sepx1, Sepw1, Sep15, and SPS2) in chicken brain tissues and embryonic neurocytes was shown in Supplementary Tables 1 and 2, respectively. Twenty-five selenoproteins in the Pb group were significantly lower (*P* < 0.05) than those in the control, Se, and Se/Pb groups at all the time points in the brain tissues and embryonic neurocytes except GPx1, Txnrd3, Dio3, SelH, and SelI for 30 days; Txnrd3, SelT, and SelO for 60 days; GPx1, GPx3, Dio2, Dio3, and SelS for 90 days; GPx1, GPx2, GPx3, Txnrd1, Dio1, Dio2, and SelO for 12 hours; GPx1, GPx4, Txnrd1, Dio1, Dio2, SelS, SelH, SelM, SelU, SelO, Sepn1, Sepp1, Sepx1, Sepw1, and SPS2 for 24 hours; GPx1, GPx2, GPx4, Txnrd2, Txnrd3, Dio1, Dio2, SelS, SelH, SelU, SelI, SelO, and Sepn1 for 36 hours; and GPx1, GPx2, Txnrd3, Dio1, SelS, SelH, SelM, SelU, SelO, Sepn1, Sepp1, Sepw1, and SPS2 for 48 hours. The twenty-five selenoproteins in the Se/Pb group were significantly lower (*P* < 0.05) than those in the control and Se groups at all the time points in the brain tissues and embryonic neurocytes.

**Table 1:** Principle component analysis results of twenty-five selenoproteins and five apoptosis-related genes in the chicken brain tissues

Component	Initial eigenvalues			Extraction sums of squared		
	Total	% of variance	Cumulative %	Total	% of variance	Cumulative %
1	27.792	92.641	92.641	27.792	92.641	92.641
2	0.593	1.978	94.619	0.593	1.978	94.619
3	0.401	1.336	95.956			
4	0.196	0.653	96.608			
5	0.166	0.553	97.161			
6	0.130	0.434	97.595			
7	0.111	0.371	97.966			
8	0.099	0.329	98.295			
9	0.085	0.283	98.578			
10	0.071	0.238	98.815			
11	0.065	0.216	99.031			
12	0.052	0.175	99.206			
13	0.043	0.143	99.349			
14	0.037	0.125	99.474			
15	0.034	0.112	99.586			
16	0.026	0.088	99.674			
17	0.025	0.085	99.759			
18	0.019	0.062	99.821			
19	0.014	0.046	99.867			
20	0.010	0.034	99.901			
21	0.007	0.024	99.926			
22	0.006	0.021	99.946			
23	0.006	0.019	99.965			
24	0.004	0.013	99.978			
25	0.002	0.007	99.986			
26	0.002	0.006	99.992			
27	0.001	0.004	99.996			
28	0.001	0.002	99.998			
29	0.000	0.001	99.999			
30	0.000	0.001	100.000			

**Table 2:** Principle component analysis results of twenty-five selenoproteins and five apoptosis-related genes in the chicken embryonic neurocytes

Component	Initial eigenvalues			Extraction sums of squared		
	Total	% of variance	Cumulative %	Total	% of variance	Cumulative %
1	28.063	93.543	93.543	28.063	93.543	93.543
2	0.484	1.612	95.156	0.484	1.612	95.156
3	0.332	1.107	96.263			
4	0.199	0.663	96.926			
5	0.157	0.525	97.450			
6	0.142	0.474	97.924			
7	0.101	0.335	98.259			
8	0.090	0.301	98.560			
9	0.072	0.239	98.800			
10	0.056	0.187	98.987			
11	0.051	0.170	99.156			
12	0.046	0.153	99.309			
13	0.037	0.124	99.433			
14	0.033	0.110	99.543			
15	0.028	0.094	99.638			
16	0.024	0.081	99.718			
17	0.020	0.068	99.786			
18	0.016	0.052	99.839			
19	0.013	0.044	99.883			
20	0.012	0.039	99.921			
21	0.008	0.027	99.949			
22	0.006	0.020	99.968			
23	0.003	0.010	99.978			
24	0.003	0.010	99.988			
25	0.002	0.006	99.994			
26	0.001	0.003	99.997			
27	0.000	0.002	99.999			
28	0.000	0.001	99.999			
29	0.000	0.000	100.000			
30	2.849E-5	9.498E-5	100.000			

In the brain tissues for the Pb group, GPx1, Txnrd1, Txnrd2, Txnrd3, Dio1, Dio3, SelH, SelI, Selpb, Sepn1, Sepp1, Sepx1, Sepw1, and Sep15 for 60 and 90 days were significantly lower (*P* < 0.05) than those for 30 days. GPx2, SelT, and SelO for 90 days were significantly lower (*P* < 0.05) than those for 30 and 60 days. GPx4 and SelM decreased significantly (*P* < 0.05) with the increase of treatment time. In the embryonic neurocytes for the Pb group, Txnrd1 decreased significantly (*P* < 0.05) with the increase of treatment time for 12, 24, and 36 hours. Dio1, Dio3, and SelK decreased significantly (*P* < 0.05) with the increase of treatment time for 24, 36, and 48 hours. Dio2, SelS, and SelM for 36 and 48 hours were significantly lower (*P* < 0.05) than those for 12 and 24 hours. SelU decreased significantly (*P* < 0.05) with the increase of treatment time. Sepp1 for 24, 36, and 48 hours was significantly lower (*P* < 0.05) than that for 12 hours.

### SOD and GPx activities, and MDA content in chicken brain tissues and embryonic neurocytes

**Figure 3:**
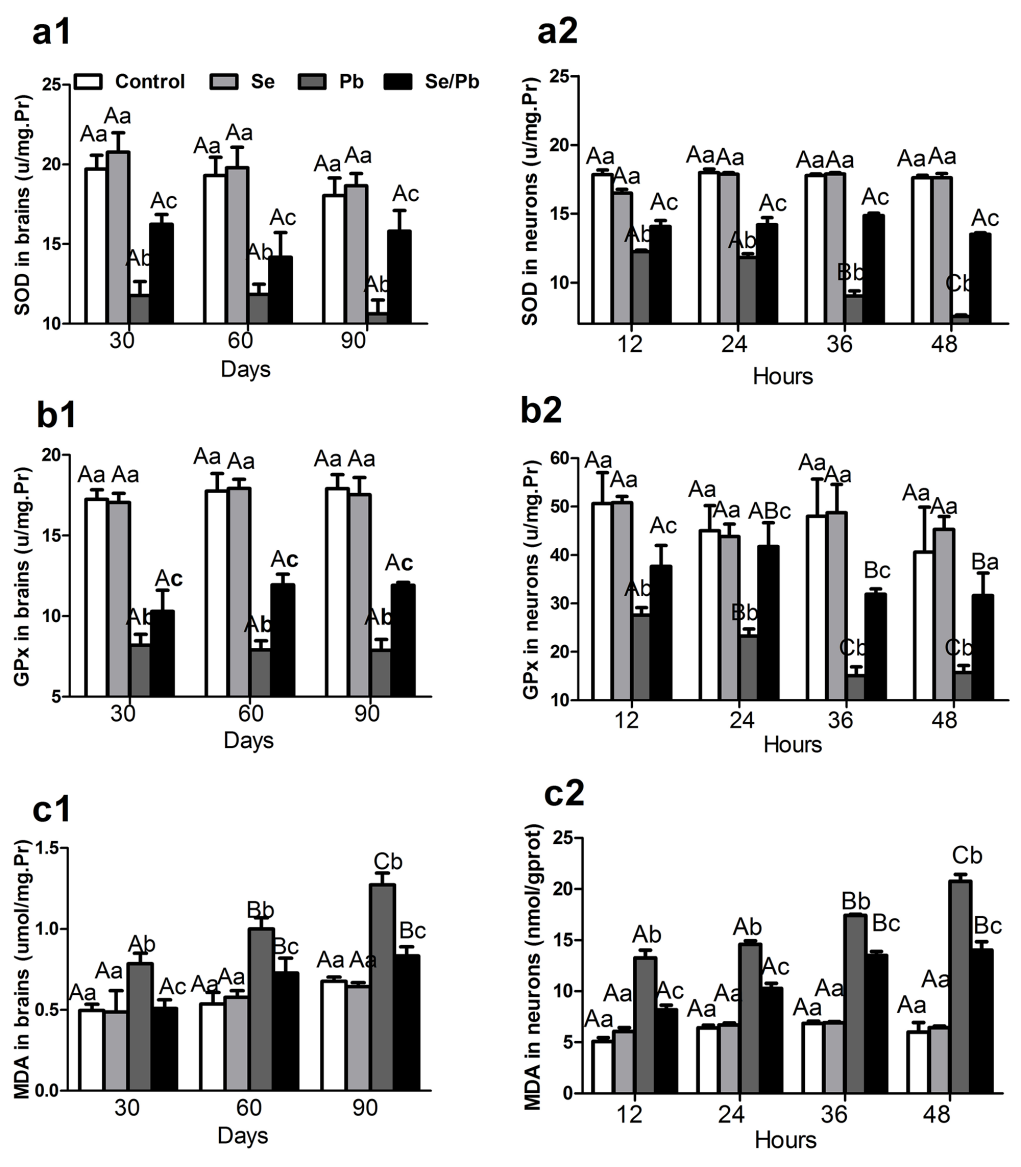
SOD and GPx activities, MDA content in the chicken brain tissues and embryonic neurocytes **(a)**: SOD activities; **(b)**: GPx activities; **(c)**: MDA content **(1)**: brain tissues; **(2)** embryonic neurocytes. *Different lowercase letters* indicate that there were significant differences (*P* < 0.05) among all groups at the same time point. *Different uppercase letters* indicate that there were significant differences (*P* < 0.05) among different time points in the same group.

Oxidative stress in chicken brain tissues and embryonic neurocytes was evaluated by determining SOD and GPx activities, and MDA content. As shown in Figure 3, SOD (Figure 3a1 and 3a2) and GPx (Figure 3b1 and 3b2) activities in the Pb group decreased significantly (*P* < 0.05) compared with those in the control, Se, and Se/Pb groups at all the time points in the brain tissues and embryonic neurocytes. SOD and GPx activities in the Se/Pb group decreased significantly (*P* < 0.05) compared with those in the control and Se groups at all the time points in the brain tissues and embryonic neurocytes except GPx activity in the embryonic neurocytes for 24 hours. MDA (Figure 3c1 and 3c2) content in the Pb group increased significantly (*P* < 0.05) compared with that in the control, Se, and Se/Pb groups at all the time points in the brain tissues and embryonic neurocytes. MDA content in the Se/Pb group increased significantly (*P* < 0.05) compared with that in the control and Se groups at all the time points.

In the embryonic neurocytes for the Pb group, SOD activity for 36 and 48 hours decreased significantly (*P* < 0.05) compared with that for 12 and 24 hours. SOD activity for 48 hours decreased significantly (*P* < 0.05) compared with that for 36 hours. GPx activity for 24 hours decreased significantly (*P* < 0.05) compared with that for 12 hours. GPx activity for 36 and 48 hours decreased significantly (*P* < 0.05) compared with that for 24 hours. MDA content increased significantly (*P* < 0.05) with the increase of treatment time. MDA content for 36 and 48 hours increased significantly (*P* < 0.05) compared with that for 12 and 24 hours. MDA content for 48 hours increased significantly (*P* < 0.05) compared with that for 36 hours.

### Relative mRNA and protein expression of five apoptosis-related genes in chicken brain tissues and embryonic neurocytes

**Figure 4:**
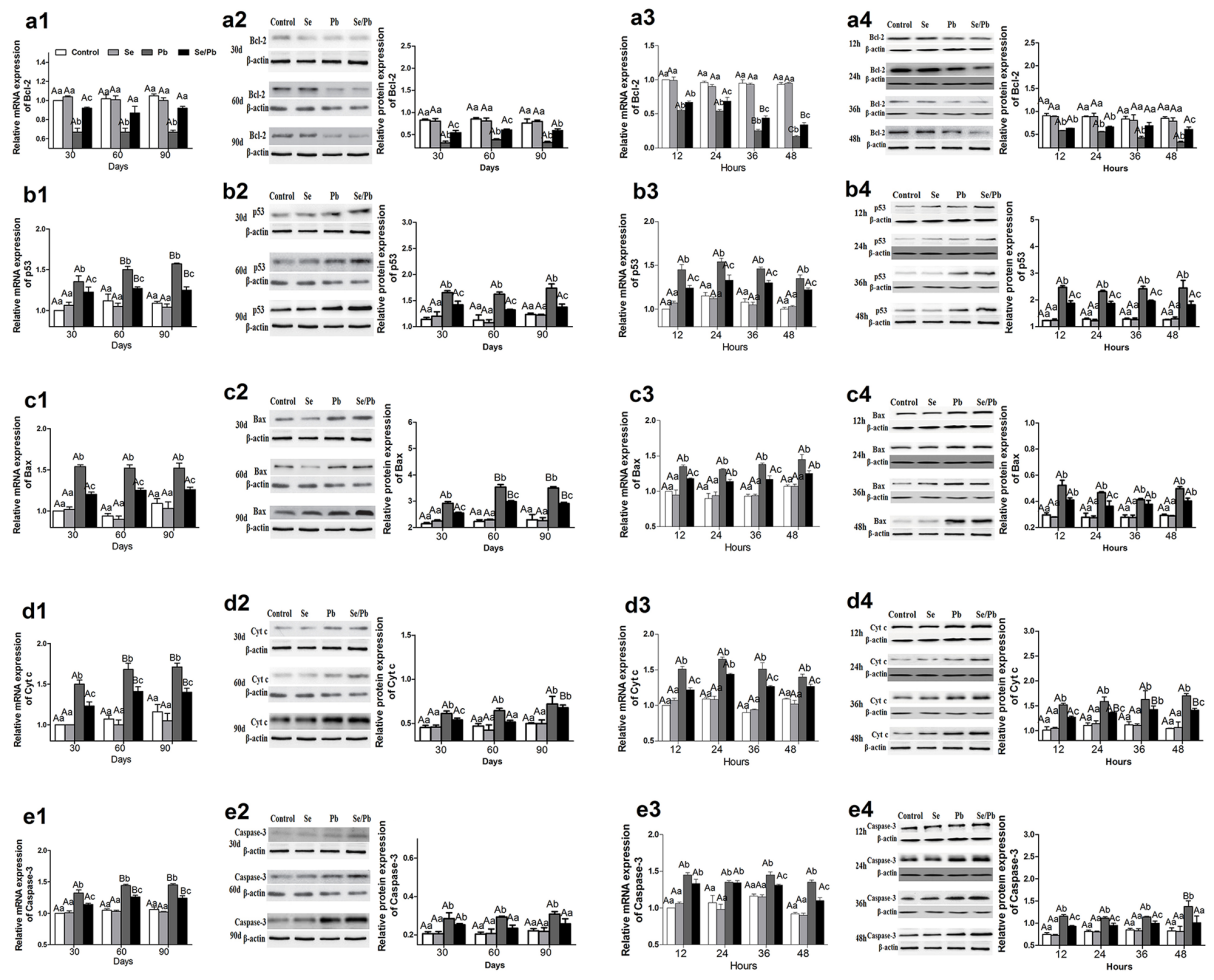
Relative mRNA and protein expression of five apoptosis-related genes in the chicken brain tissues and embryonic neurocytes **(a)**: Bcl-2 expression; **(b)**: p53 expression; **(c)**: Bax expression; **(d)**: Cyt c expression; **(e)**: Caspase-3 expression **(1)**: mRNA expression in brain tissues; **(2)**: protein expression in brain tissues; **(3)**: mRNA expression in embryonic neurocytes; **(4)**: protein expression in embryonic neurocytes. *Different lowercase letters* indicate that there were significant differences (*P* < 0.05) among all groups at the same time point. *Different uppercase letters* indicate that there were significant differences (*P* < 0.05) among different time points in the same group.

To investigate toxic effect of excess Pb on apoptosis and alleviative effect of Se on Pb-induced apoptosis, mRNA and protein expression of five apoptosis-related genes (Bcl-2, p53, Bax, Cyt c, and Caspase-3) was examined in chicken brain tissues and embryonic neurocytes. Bcl-2 (Figure 4a1-4a4) in the Pb group was significantly lower (*P* < 0.05) than that in all the other groups at all the time points in the brain tissues and embryonic neurocytes except mRNA expression for 12 hours; and protein expression for 90 days, 12 hours, and 24 hours in the Se/Pb group. Bcl-2 in the Se/Pb group was significantly lower (*P* < 0.05) than that in the control and Se groups at all the time points except mRNA expression for 60 and 90 days; and protein expression for 36 hours. p53 (Figure 4b1-4b4), Bax (Figure 4c1-4c4), Cyt c (Figure 4d1-4d4), and Caspase-3 (Figure 4e1-4e4) in the Pb group were significantly higher (*P* < 0.05) than those in all the other groups at all the time points except Bax mRNA expression for 24 and 48 hours; Bax protein expression for 12, 36, and 48 hours; Cyt c mRNA expression for 24 hours, Cyt c protein expression for 90 days and for 36 hours; Caspase-3 mRNA expression for 12 and 24 hours; Caspase-3 protein expression for 30 days in the Se/Pb group. p53, Bax, Cyt c, and Caspase-3 in the Se/Pb group were significantly higher (*P* < 0.05) than those in the control and Se groups at all the time points except Cyt c protein expression for 60 days; Caspase-3 protein expression for 60 and 90 days, and for 48 hours.

In the brain tissues for the Pb group, mRNA expression of p53 and Cyt c and protein expression of Bax for 60 and 90 days were significantly higher (*P* < 0.05) than those for 30 days. In the embryonic neurocytes for the Pb group, Bcl-2 mRNA expression decreased significantly (*P* < 0.05) with the increase of treatment time for 24, 36, and 48 hours. Caspase-3 protein expression for 48 hours was significantly higher (*P* < 0.05) than that for 12, 24, and 36 hours.

### Multivariate correlation analysis

Pearson's r correlation coefficient was used for multivariate correlation analysis among all twenty-five selenoproteins and five apoptosis-related genes in chicken brain tissues and embryonic neurocytes, respectively. The results showed that there were significantly positive relationships at the 0.01 level among twenty-five selenoproteins; among four apoptosis-related genes (p53, Bax, Cyt c, and Caspase-3); and between twenty-five selenoproteins and Bcl-2 in the brain tissues and embryonic neurocytes. There were significantly negative relationships at the 0.01 level between twenty-five selenoproteins and four apoptosis-related genes (p53, Bax, Cyt c, and Caspase-3) in the brain tissues and embryonic neurocytes.

### PCA

**Figure 5:**
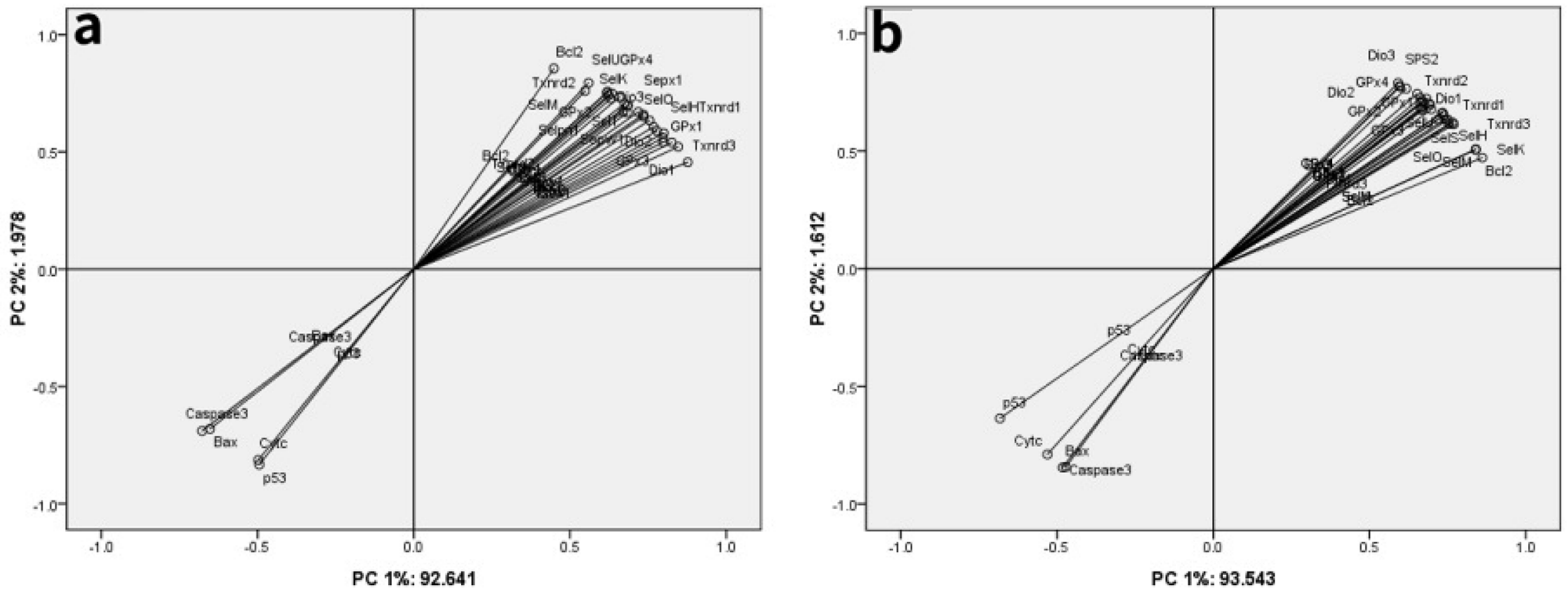
Principle component analysis among twenty-five selenoproteins and five apoptosis-related genes in the chicken brain tissues and embryonic neurocytes **(a)** brain tissues; **(b)** embryonic neurocytes.

Twenty-five selenoproteins and five apoptosis-related genes were used for PCA in chicken brain tissues and embryonic neurocytes (Tables 1 and 2 and Figure 5). The results showed that all the parameters focused on the first two principal components. In the brain tissues, the first two principal components reflected 94.619% original data. Principal component (PC) 1 and PC 2 accounted for 92.641% and 1.978% of explained variances, respectively (Table 1). In the embryonic neurocytes, the first two principal components reflected 95.155% original data. PC 1 and PC 2 accounted for 93.543% and 1.612% of explained variances, respectively (Table 2). Twenty-five selenoproteins and Bcl-2 belonged to PC 1 in the brain tissues (Figure 5a) and embryonic neurocytes (Figure 5b). p53, Bax, Cyt c, and Caspase-3 belonged to PC 2 in the brain tissues (Figure 5a) and embryonic neurocytes (Figure 5b).

## DISCUSSION

Pb can induce neuronal apoptosis such as Pb-induced apoptosis in rat neuronal [9]. Se mainly performs its function through Se-containing proteins [15]. Se can protect against oxidative damage [27, 28] via selenoproteins [28]. Se had a protective effect for Cd-induced oxidative stress and apoptosis via mitochondria pathway in mouse kidneys [14]. Yao et al. found that Sepw1 served as an antioxidant [29] and protected embryonic myoblasts against apoptosis induced by hydrogen peroxide (H_2_O_2_) in chickens [30]. The decrease of GPx1, GPx2, GPx3, GPx4, Txnrd1, SelT, SelK, SelH, SelM, and SelI mRNA expression caused oxidative stress in swine peripheral blood neutrophils *in vitro* [31]. The decrease of SelW increased MDA content, and caused oxidative stress in chicken myoblasts and embryos [32]. Se can alleviate the decrease of selenoproteins, and oxidative stress caused by heavy metals in animals. Se alleviated Cd-caused the decrease of GPx4 mRNA expression, and oxidative stress in rat testes [17], and the decrease of SelT, SelK, and SelS mRNA expression in chicken splenic lymphocytes *in vitro* [18]. Se alleviated Pb-caused the decrease of GPx1, GPx2, GPx4, Txnrd2, Txnrd3, Dio1, Dio2, SelT, SelK, SelS, SelM, SelU, SelI, SelO, Sepn1, Sepx1, and Sep15 mRNA expression in chicken meniscus cartilages; the decrease of GPx2, GPx3, GPx4, Txnrd1, Txnrd2, Dio2, Dio3, SelT, SelK, SelH, SelM, SelI, SelO, Selpb, Sepn1, Sepx1, Sep15, and SPS2 mRNA expression in chicken sword cartilages; and oxidative stress in chicken meniscus cartilages and sword cartilages [22]. Our results were consistent with the above studies. We found that Se alleviated Pb-caused the decrease of GPx1, GPx2, GPx3, GPx4, Txnrd1, Txnrd2, Txnrd3, Dio1, Dio2, Dio3, SelT, SelK, SelS, SelH, SelM, SelU, SelI, SelO, Selpb, Sepn1, Sepp1, Sepx1, Sepw1, Sep15, and SPS2 mRNA expression in the chicken brain tissues and embryonic neurocytes. Our results indicated that Se alleviated Pb-induced oxidative stress via increasing selenoproteins in the chicken nervous tissues *in vivo* and *in vitro*. Moreover, we also found that there was a time-dependent effect on Pb-caused the decrease of GPx4 and SelM mRNA expression in the brain tissues and the decrease of SelU mRNA expression in the embryonic neurocytes. In addition, multivariate correlation analysis showed that there were positive relationships among the twenty-five selenoproteins in the chicken brain tissues and embryonic neurocytes. Huang et al. [26] also found that there were positive relationships among twenty-five selenoproteins in chicken testes*.* PCA revealed that the twenty-five selenoproteins belonged to PC 1 in the chicken brain tissues and embryonic neurocytes. Results of multivariate correlation analysis and PCA were confirmed the reliability of our results. The mechanisms of strong positive relationships among selenoproteins need to be further studied.

Excess Pb can cause oxidative stress, which is one possible mechanism of negative effect of Pb on organisms [33]. Oxidative stress can disrupt prooxidant/antioxidant balance in mammalian cells [33]. Pb caused oxidative stress and induced apoptosis in rat proximal tubular cells [34]. SOD, GPx, and MDA are antioxidant indexes. Pb decreased SOD and GPx activities, increased MDA content, and caused oxidative stress in mouse kidneys [35]. Arsenic exposure induced neurotoxicity and oxidative stress by the decrease of GPx activity, the increase of MDA content, and the changes of histopathology in chicken brain tissues [36]. Se alleviated Cd-caused the decrease of SOD and GPx activities, the increase of MDA content, and oxidative stress in chicken kidneys [37]. In accordance with the above researches, our study indicated that Se alleviated Pb-caused the decrease of SOD and GPx activities, the increase of MDA content, and oxidative stress in the chicken brain tissues and embryonic neurocytes. In addition, in our experiment, there was a time-dependent effect on Pb-induced the increase of MDA content in the chicken brain tissues. Schlorff et al. [38] also found that ethanol increased MDA content in a time-dependent manner in rat plasmas.

Apoptosis is a physiological cell suicide program that is essential for regulation of development and maintenance of homeostasis [39]. Oxidative stress induced apoptosis in rat proximal tubular cells [40]. Bcl-2 is known as an anti-apoptotic protein which protects cells from apoptosis [41]. Bcl-2 can inhibit the release of Cyt c from mitochondria and protect apoptosis induced by oxidative stress [41]. Pro-apoptotic proteins such as Bax can promote apoptosis [42]. p53 accumulates during apoptosis process [43, 44]. p53 can promote neuronal apoptosis by increasing Bax transcription [45]. The induction of p53 leads to Cyt c release [46]. The release of Cyt c activates Caspase-3 [10]. The activation of Caspase-3 induces apoptosis [10]. Our results also demonstrated the above mechanism. We found that Pb decreased mRNA and protein expression of Bcl-2; increased mRNA and protein expression of p53, Bax, Cyt c, and Caspase-3; and induced apoptosis in the chicken brain tissues and embryonic neurocytes via mitochondrial pathway. In our results, morphological changes also demonstrated that Pb caused apoptosis in the chicken brain tissues and embryonic neurocytes. Other researches were similar with our research results. Liu et al. [11] found that Pb caused ultrastructural changes and apoptosis in rat proximal tubular cells. Zhang et al. [47] found that molybdenum and Cd changed ultrastructural structure and induced apoptosis in duck spleens. Nickel chloride decreased Bcl-2 mRNA expression, increased Bax, Cyt c, and Caspase-3 mRNA expression, and induced apoptosis via mitochondrial pathway in chicken bursa of Fabricius [48]. Sodium fluoride decreased Bcl-2 protein expression, increased Bax and Caspase-3 protein expression, and induced apoptosis in a time- and dose-dependent manner in mice splenic lymphocytes via mitochondria pathway [49]. Flora et al. [9] demonstrated that Pb decreased Bcl-2 protein expression, increased p53, Bax, Cyt c, and Caspase-3 protein expression, and induced apoptosis in rat brains. Pb caused the disruption of mitochondrial structure, the release of Cyt c, the activation of Caspase-3, and apoptosis in rat proximal tubular cells [11]. In addition, multivariate correlation analysis showed that there were positive relationships among four apoptosis-related genes (p53, Bax, Cyt c, and Caspase-3). There were negative relationships between Bcl-2 and four apoptosis-related genes (p53, Bax, Cyt c, and Caspase-3) in the chicken brain tissues and embryonic neurocytes. The PCA also revealed that Bcl-2 and four apoptosis-related genes (p53, Bax, Cyt c, and Caspase-3) belonged to different components in the chicken brain tissues and embryonic neurocytes. In our study, we also found that there were positive relationships between twenty-five selenoproteins and Bcl-2; and there were negative relationships between twenty-five selenoproteins and four apoptosis-related genes (p53, Bax, Cyt c, and Caspase-3) in the chicken brain tissues and embryonic neurocytes. The PCA also revealed that twenty-five selenoproteins and Bcl-2 belonged to PC 1. Twenty-five selenoproteins are closely related with Bcl-2. Other researches have also found similar mechanism. Yao et al. [30] reported that H_2_O_2_ caused the decrease of SelW and Bcl-2 protein expression in chicken myoblasts. Wang et al. [50] found that there were strong positive relationships between Bcl-2 and ten selenoproteins (GPx3, GPx4, Txnrd1, Txnrd2, Txnrd3, Dio1, SelS, SelI, Selpb, and Sep15); there were strong negative relationships between Cyt c and eight selenoproteins (GPx4, Dio1, SelS, SelH, SelI, Selpb, Sepp1, and Sep15); and there were strong negative relationships between Caspase-3 and ten selenoproteins (GPx3, GPx4, Dio1, Txnrd1, Txnrd2, Txnrd3, SelS, SelI, Selpb, and SPS2) in chick embryonic vascular smooth muscle cells. The mechanisms of positive relationships between twenty-five selenoproteins (GPx1, GPx2, GPx3, GPx4, Txnrd1, Txnrd2, Txnrd3, Dio1, Dio2, Dio3, SelT, SelK, SelS, SelH, SelM, SelU, SelI, SelO, Selpb, Sepn1, Sepp1, Sepx1, Sepw1, Sep15, and SPS2) and Bcl-2, and negative relationships between twenty-five selenoproteins and the four apoptosis-related genes (p53, Bax, Cyt c, and Caspase-3) need to be further studied. Se can alleviate apoptosis induced by heavy metals via mitochondria pathway [14]. Se was found to prevent Cd-induced apoptosis mediated by oxidative stress via ameliorating mitochondrial dysfunction in porcine renal epithelial cells [51]. Wang et al. [14] found that Se alleviated Cd-caused the decrease of Bcl-2 protein expression, the increase of Bax protein expression, and apoptosis via mitochondria pathway in mouse kidneys. Se alleviated Cd-caused the decrease of Bcl-2 mRNA expression; the increase of p53, Bax, Cyt c, and Caspase-3 mRNA expression; and apoptosis via mitochondrial pathway in chicken splenic lymphocytes *in vitro* [21]. Consistent with these reports, our results indicated that Se alleviated Pb-caused the decrease of Bcl-2 mRNA and protein expression, the increase of p53, Bax, Cyt c, and Caspase-3 mRNA and protein expression, and apoptosis via mitochondrial pathway in the chicken brain tissues and embryonic neurocytes. Moreover, our morphological examination revealed that Se alleviated Pb-caused apoptosis in the chicken brain tissues and embryonic neurocytes.

In summary, our data indicated that Pb caused morphological changes; decreased SOD and GPx activities; increased MDA content; decreased GPx1, GPx2, GPx3, GPx4, Txnrd1, Txnrd2, Txnrd3, Dio1, Dio2, Dio3, SelT, SelK, SelS, SelH, SelM, SelU, SelI, SelO, Selpb, Sepn1, Sepp1, Sepx1, Sepw1, Sep15, and SPS2 mRNA expression, Bcl-2 mRNA and protein expression; and increased p53, Bax, Cyt c, and Caspase-3 mRNA and protein expression in the chicken brain tissues and embryonic neurocytes. Se alleviated Pb-caused all of the above changes in the chicken brain tissues and embryonic neurocytes. There were time-dependent manners on GPx4, SelM, and MDA in the chicken brain tissues; and on SelU in the chicken embryonic neurocytes. Consistent with researches in mammals, our data demonstrated that Se alleviated Pb-induced apoptosis in the chicken nervous tissues via mitochondrial pathway.

## MATERIALS AND METHODS

### Animal model and tissue samples

One hundred and eighty 1-day-old healthy Hyline male chickens were fed a standard commercial diet (containing 0.49 mg/kg Se) and drinking water. Chickens were randomly divided into four groups (forty-five chickens per group) at 8 days of age. The control group was fed the standard commercial diet and drinking water. The Se group was fed sodium selenite (Na_2_SeO_3_, analytical reagent grade, Tianjinzhiyuan Chemical Reagent Co. Ltd., Tianjin, China) added to the standard commercial diet at 1 mg/kg Se and drinking water. The Pb group was fed the standard commercial diet and lead acetate ((CH_3_COO)_2_Pb, analytical reagent grade, Tianjinzhiyuan Chemical Reagent Co. Ltd., Tianjin, China) added to drinking water at 350 mg/L Pb, according to median lethal dose (1739.3 mg/kg body weight) for Pb in chickens [52] and the need for the chicken experiments in toxicology [53]. The Se/Pb group was fed Na_2_SeO_3_ added to the standard commercial diet at 1 mg/kg Se and (CH_3_COO)_2_Pb added to drinking water at 350 mg/L Pb. Food and water were provided ad libitum. The chickens were fed in the Laboratory Animal Center, Animal Medical College, Northeast Agricultural University (Harbin, China). All procedures used in this experiment were approved by the Northeast Agricultural University's Institutional Animal Care and Use Committee under the approved protocol number SRM-06.

Fifteen chickens per group were randomly selected and euthanized on the 30^th^, 60^th^, and 90^th^ days of the experiment, respectively. Next, brain tissues were quickly removed and rinsed with ice-cold sterile deionized water. Some samples were immediately frozen in liquid nitrogen and stored at -80 °C to determine mRNA expression of twenty-five selenoproteins (GPx1, GPx2, GPx3, GPx4, Txnrd1, Txnrd2, Txnrd3, Dio1, Dio2, Dio3, SelT, SelK, SelS, SelH, SelM, SelU, SelI, SelO, Selpb, Sepn1, Sepp1, Sepx1, Sepw1, Sep15, and SPS2); and mRNA and protein expression of five apoptosis-related genes (Bcl-2, p53, Bax, Cyt c, and Caspase-3). Some samples were immediately homogenized to detect SOD and GPx activities, and MDA content. The remaining samples were stored in 2.5% glutaraldehyde phosphate buffered saline (PBS, 0.1 M phosphate buffer with 0.85% sodium chloride, v/v, pH 7.2) to observe ultrastructure.

### Chicken embryonic neurocytes

Fertilized eggs were hatched in artificial hatching incubator (38 °C, 50% humidity) for 6-9 days. Embryos were carefully removed from eggs under aseptic conditions. Brain tissues were obtained and put into a sterile Petri dish containing PBS and washed twice at room temperature. After meninges and vessels being removed, embryos were cut into small pieces (approximately 1-2 mm^3^) and transferred into a sterile Petri dish containing PBS with 0.125% trypsin (pH 7.2, Sigma-Aldrich, USA) at 37 °C in an atmosphere of 5% CO_2_ for 15 minutes. The Petri dish was brought out from the incubator and mixed complete Dulbecco's modified Eagle's medium (DMEM) culture solution (Gibco, USA) [50 mL of fetal bovine serum (FBS, Gibco, USA), 5 mL of penicillin/streptomycin (Sigma, USA), and 500 mL of DMEM] was put into the Petri dish. The Petri dish was rested for 2 minutes. Supernatant liquid was poured out. Neuronal cell suspension was washed twice in the same culture medium. The neuronal cell suspension was filtered with a sterile stainless steel mesh with 400 mm pore size. Cell density was adjusted to 5 × 10^6^ cells/mL with the same culture medium. Cell viability was detected using trypan blue exclusion test and was above 95%. Na_2_SeO_3_ at 10^−7^ mol/L Se in the Se group, (CH_3_COO)_2_Pb at 10^−6^ mol/L Pb in the Pb group, and Na_2_SeO_3_ at 10^−7^ mol/L Se and (CH_3_COO)_2_Pb at 10^−6^ mol/L Pb in the Se/Pb group were immediately added to the culture medium, respectively. Embryonic neurocytes in the control, Se, Pb, and Se/Pb groups were cultured in poly-l-lysine (Sigma-Aldrich, USA)-coated (0.1%, 4 hours) cell culture plates for 12, 24, 36, and 48 hours. Supernatant was poured out. 1 mL of RNAiso Plus was added into the culture plates. The embryonic neurocytes were used to perform cell morphology, mRNA and protein expression of five apoptosis-related genes, mRNA expression of twenty-five selenoproteins, SOD and GPx activities, and MDA content.

### Determination of IC50 of Pb in chicken embryonic neurocytes

The IC50 of chicken embryonic neurocytes in the Pb and Se/Pb groups for 48 hours was measured using cell counting kit-8 produced by Nanjing Jiancheng Bioengineering Institute (Nanjing, China) according to the manufacturer's instructions. In the Pb group, Pb concentration was 2, 4, 6, 8, 10, 12, 14, 16, and 18 × 10^−7^ mol/L, respectively. In the Se/Pb group, Pb concentration was 2, 4, 6, 8, 10, 12, 14, 16, and 18 × 10^−7^ mol/L, respectively; and Se concentration was 10 × 10^−8^ mol/L.

### Ultrastructure

Brain tissue specimens were embedded in araldite (Sigma-Aldrich, USA). Ultrathin section was stained with Mg-uranyl acetate and lead citrate (Sigma-Aldrich, USA) for transmission electron microscope observation. Specimen handling procedure was performed following the method described in our previous study [54].

### Cell morphology

Apoptosis observation of chicken embryonic neurocytes was performed using acridine orange (Amrisco, USA) and ethidium bromide (Sigma-Aldrich, USA) double staining. Cell staining process was performed following the method described in our previous study [55].

### SOD and GPx activities, and MDA content

SOD and GPx activities, and MDA content were examined using SOD, GPx, and MDA detection kits according to the instructions of the reagent company (Nanjing Jiancheng Bioengineering Institute, Nanjing, China).

### Relative mRNA expression of five apoptosis-related genes and twenty-five selenoproteins

Primer sequences (Table 3) of β-actin; five apoptosis-related genes (Bcl-2, p53, Bax, Cyt c, and Caspase-3); and twenty-five selenoproteins (GPx1, GPx2, GPx3, GPx4, Txnrd1, Txnrd2, Txnrd3, Dio1, Dio2, Dio3, SelT, SelK, SelS, SelH, SelM, SelU, SelI, SelO, Selpb, Sepn1, Sepp1, Sepx1, Sepw1, Sep15, and SPS2) published in GenBank were synthesized by Invitrogen Biotechnology Co. Ltd., Shanghai, China. β-actin was used as an internal reference gene. Total RNA was extracted using RNAiso Plus reagent (Takara, Japan), and then RNA was reverse transcribed in 40 μL of reaction mixture according to the manufacturer's instructions (Invitrogen, USA).

**Table 3:** Primers used for quantitative real-time PCR

Gene	Serial number	Primer sequence	
GPx1	NM_001277853.1	F: 5’-ACGGCGCATCTTCCAAAG-3’	R: 5’-TGTTCCCCCAACCATTTCTC-3’
GPx2	NM_001277854.1	F: 5’-ATCGCCAAGTCCTTCTACGA-3’	R: 5’-ACGTTCTCGATGAGGACCAC-3’
GPx3	NM_001163232.2	F: 5’-CCTGCAGTACCTCGAACTGA-3’	R: 5’-CTTCAGTGCAGGGAGGATCT-3’
GPx4	AF498316.2	F: 5’-CTTCGTCTGCATCATCACCAA-3’	R: 5’-TCGACGAGCTGAGTGTAATTCAC-3’
Txnrd1	NM_001030762.2	F: 5’-TACGCCTCTGGGAAATTCGT-3’	R: 5’-CTTGCAAGGCTTGTCCCAGTA-3’
Txnrd2	NM_001122691.1	F: 5’-GCTCTTAAAGATGCCCAGCACTAC-3’	R: 5’-GAACAGCTTGAGCCATCACAGA-3’
Txnrd3	NM_001122777.1	F: 5’-CCTGGCAAAACGCTAGTTGTG-3’	R: 5’-CGCACCATTACTGTGACATCTAGAC-3’
Dio1	NM_001097614.1	F: 5’-GCGCTATACCACAGGCAGTA-3’	R: 5’-GGTCTTGCAAATGTCACCAC-3’
Dio2	NM_204114.3	F: 5’-ATTTGCTGATCACGCTTCAG-3’	R: 5’-GCTCAGAAACAGCACCATGT-3’
Dio3	NM_001122648.1	F: 5’-CTGTGCATTCGCAAGAAGAT-3’	R: 5’-GCCGACTTGAAGAAGTCCAG-3’
SelT	NM_001006557.3	F: 5’-AGGAGTACATGCGGGTCATCA-3’	R: 5’-GACAGACAGGAAGGATGCTATGTG-3’
SelK	NM_001025441.2	F: 5’-ATGACGACCACCCTCACGAT-3’	R: 5’-CCAGCGTTAACCGGAATGAT-3
SelS	NM_173120.2	F: 5’-GCCTGCGTCGCCATCTATCTCA-3’	R: 5’-TTCTGCCTTCGCTTCTGTTCTTCAA-3’
SelH	NM_001277865.1	F: 5’-CATCGAGCACTGCCGTAG-3’	R: 5’-GACACCTCGAAGCTGTTCCT-3’
SelM	NM_001277859.1	F: 5’-AAGAAGGACCACCCAGACCT-3’	R: 5’-GCTGTCCTGTCTCCCTCATC-3’
SelU	NM_001193518.1	F: 5’-GATGCTTTCAGGCTTCTTCC-3’	R: 5’-CTGTCTTCCTGCTCCAATCA-3’
SelI	NM_001031528.2	F: 5’-TGCCAGCCTCTGAACTGGAT-3’	R: 5’-TGCAAACCCAGACATCACCAT-3’
SelO	NM_001115017.1	F: 5’-CCAGCGTTAACCGGAATGAT-3’	R: 5’-GCCTACAGAATGGATCCAACTGA-3’
Selpb	XM_003641687.2	F: 5’-AGGCCAACAGTACCATGGAG-3’	R: 5’-GTGGTGAGGATGGAGATGGT-3’
Sepn1	NM_001114972.1	F: 5’-CAGGATCCATGCTGAGTTCCA-3’	R: 5’-GAGAGGACGATGTAACCCGTAAAC-3’
Sepp1	NM_001031609	F: 5’-CCAAGTGGTCAGCATTCACATC-3’	R: 5’-ATGACGACCACCCTCACGAT-3’
Sepx1	NM_001135558.2	F: 5’-TGGCAAGTGTGGCAATGG-3’	R: 5’-GAATTTGAGCGAGCTGCTGAAT-3’
Sepw1	NM_001166327.1	F: 5’-TGGTGTGGGTCTGCTTTACG-3’	R: 5’-CCAAAGCTGGAAGGTGCAA-3’
Sep15	NM_001012926.2	F: 5’-ACTTGGCTTCTCCAGTAACTTGCT-3’	R: 5’-GCCTACAGAATGGATCCAACTGA-3’
SPS2	BM489698.1	F: 5’-CGTTGGGTATCGGAACTGAC-3’	R: 5’-CGTCCACCAGAGGGTAGAAA-3’
Bcl-2	NM_205339.1	F: 5’-ATCGTCGCCTTCTTCGAGTT-3’	R: 5’-ATCCCATCCTCCGTTGTCCT-3’
p53	NM_205264.1	F: 5’-GAGATGCTGAAGGAGATCAATGAG-3’	R: 5’-GTGGTCAGTCCGAGCCTTTT-3’
Bax	FJ977571.1	F: 5’-GATGAAGCCACCCAGCAGTA-3’	R: 5’-TGGATTCTCACAGTAGGAGGATG-3’
Cyt c	K02303.1	F: 5’-AGGCAAGCACAAGACTGGA-3’	R: 5’-CTGACTATCACCAAGAACCACC-3’
Caspase-3	NM_204725.1	F: 5’-CATCTGCATCCGTGCCTGA-3’	R: 5’-CTCTCGGCTGTGGTGGTGAA-3’
β-actin	L08165	F: 5’-CCGCTCTATGAAGGCTACGC-3’	R: 5’-CTC TCG GCT GTG GTGGTG AA-3’

Quantitative real-time polymerase chain reaction (PCR) was performed using SYBR^®^ Premix Ex Taq^TM^ II (Takara, Japan) with the Applied Biosystems PRISM 7500 real-time PCR system according to the manufacturer's instructions (Applied Biosystems, Foster, USA). The experiment was repeated three times for each sample. Relative mRNA abundance for each gene was calculated according to the method of 2^−ΔΔCt^ [56].

### Relative protein expression of five apoptosis-related genes

Relative protein expression of five apoptosis-related genes (Bcl-2, p53, Bax, Cyt c, and Caspase-3) was measured using Western blot assay followed the method described in previous study (28). The first antibody (1:100), secondary antibody (1:1000), monoclonal β-actin antibody (1:1000), and goat antimouse IgG (1:1000) of Bcl-2, p53, Bax, Cyt c, and Caspase-3 were purchased from Santa Cruz Biotechnology, USA. The signal was detected using X-ray films (TianGen Biotech Co. Ltd., Beijing, China). The optical density of each band was measured using Image VCD gel imaging system (Beijing Sage Creation Science And Technology Co. Ltd., China).

### Statistical analysis

All data were performed using SPSS for Windows (version 19; SPSS Inc., Chicago, IL, USA). Statistical comparisons for all data were performed using one-way or two-way analysis of variance (ANOVA) and verified by nonparametric Kruskal-Wallis ANOVA and Mann-Whitney *U* test. Data were expressed as the mean ± standard deviation (*n* = 5). Relative mRNA expression data of twenty-five selenoproteins and five apoptosis-related genes were used to perform multivariate correlation analysis and principal component analysis (PCA). Multivariate correlation analysis was used to measure linear correlations among determined factors. PCA was used to define the most important parameters, which could be used as key factors for individual variations.

## SUPPLEMENTARY MATERIALS FIGURES AND TABLES







## Abbreviations

Se: selenium
Pb: lead
(CH_3_COO)_2_Pb): lead acetate
Na_2_SeO_3_: sodium selenite
mRNA: messenger RNA
GPx: glutathione peroxidase
SOD: superoxide dismutase
Txnrd: thioredoxin reductases
Dio: iodothyronine deiodinases
SelT: selenoprotein T
Sepn1: selenoprotein n1
Sep15: 15-kDa selenoprotein
SPS2: selenophosphate synthetases 2
Bcl-2: B-cell lymphoma-2
MDA: malondialdehyde
Bax: Bcl-2 associated X protein
Cyt c: cytochrome c
PC 12: rat adrenal medulla pheochromocytoma cells
Cd: cadmium
IC50, 50%: inhibitory concentration
PBS: phosphate buffered saline
DMEM: dulbecco's modified eagle's medium
PCR: polymerase chain reaction
PCA: principal component analysis
ANOVA: analysis of variance
H_2_O_2_: hydrogen peroxide
