# Supplementary material for: Selenium against lead-induced apoptosis in chicken nervous tissues via mitochondrial pathway
Source: Oncotarget. 2017 Nov 20;8(64):108130–45. doi: 10.18632/oncotarget.22553 (PMC5746131; doi:10.18632/oncotarget.22553)
Supplement: Supplementary file 1 [file oncotarget-08-108130-s001.pdf]

Selenium against lead-induced apoptosis in chicken nervous tissues via mitochondrial pathway

SUPPLEMENTARY MATERIALS

Supplementary Table 1: The relative mRNA expression of twenty-five selenoproteins in the chicken brain tissues.

See Supplementary File 1

Supplementary Table 2: The relative mRNA expression of twenty-five selenoproteins in the chicken embryonic neurocytes.

See Supplementary File 2
