# Supplementary material for: Selenium against lead-induced apoptosis in chicken nervous tissues via mitochondrial pathway
Source: Oncotarget. 2017 Nov 20;8(64):108130–45. doi: 10.18632/oncotarget.22553 (PMC5746131; doi:10.18632/oncotarget.22553)
Supplement: Supplementary file 2 [file oncotarget-08-108130-s002.docx]

**Supplementary Table 1:** **The relative mRNA expression of twenty-five selenoproteins in the chicken brain tissues**

| **Selenoproteins** |  | **30^th^ day** | | | |  | | |  | | **60^th^ day** | | | | |  | |  | | **90^th^ day** | | | | |  | |
| --- | --- | --- | --- | --- | --- | --- | --- | --- | --- | --- | --- | --- | --- | --- | --- | --- | --- | --- | --- | --- | --- | --- | --- | --- | --- | --- |
|  |  | **Control** | **Se** | **Pb** | **Se/Pb** | |  | | |  | | **Control** | **Se** | **Pb** | **Se/Pb** | |  | |  | | **Control** | **Se** | **Pb** | **Se/Pb** | |  |
| GPx1 | 1.00±0.00^Aa^; 0.87±0.05^Aa^; 0.48±0.03^Ab^; 0.64±0.10^Ab^ | | | | | | | 0.94±0.01^Aa^; 0.96±0.05^Aa^; 0.19±0.01^Bb^; 0.34±0.03^Bc^ | | | | | | | | | | 0.96±0.04^Aa^; 0.90±0.06^Aa^; 0.18±0.01^Bb^; 0.28±0.02^Bb^ | | | | | | | | |
| Gpx2 | 1.00±0.00^Aa^; 0.91±0.02^Aa^; 0.42±0.05^Ab^; 0.58±0.04^Ac^ | | | | | | | 0.99±0.07^Aa^; 0.89±0.05^Aa^; 0.41±0.05^Ab^; 0.55±0.02^Ac^ | | | | | | | | | | 0.91±0.02^Aa^; 0.80±0.08^Aa^; 0.28±0.03^Bb^; 0.53±0.02^Ac^ | | | | | | | | |
| GPx3 | 1.00±0.00^Aa^; 1.00±0.02^Aa^; 0.37±0.14^Ab^; 0.70±0.04^Ac^ | | | | | | | 0.98±0.08^Aa^; 0.95±0.04^Aa^; 0.11±0.01^Ab^; 0.24±0.02^Bc^ | | | | | | | | | | 0.94±0.04^Aa^; 0.99±0.06^Aa^; 0.24±0.02^Ab^; 0.30±0.01^Bb^ | | | | | | | | |
| GPx4 | 1.00±0.00^Aa^; 1.00±0.03^Aa^; 0.39±0.02^Ab^; 0.55±0.01^Ac^ | | | | | | | 1.02±0.08^Aa^; 0.96±0.02^Aa^; 0.32±0.01^Bb^; 0.53±0.02^Ac^ | | | | | | | | | | 1.05±0.02^Aa^; 1.03±0.05^Aa^; 0.22±0.01^Cb^; 0.55±0.02^Ac^ | | | | | | | | |
| Txnrd1 | 1.00±0.00^Aa^; 0.98±0.05^Aa^; 0.59±0.03^Ab^; 0.84±0.09^Ac^ | | | | | | | 0.83±0.05^Aa^; 0.97±0.02^Aa^; 0.28±0.03^Bb^; 0.52±0.02^Bc^ | | | | | | | | | | 0.94±0.16^Aa^; 0.95±0.10^Aa^; 0.24±0.02^Bb^; 0.48±0.01^Bc^ | | | | | | | | |
| Txnrd2 | 1.00±0.00^Aa^; 1.02±0.01^Aa^; 0.51±0.02^Ab^; 0.87±0.06^Ac^ | | | | | | | 0.96±0.04^Aa^; 0.96±0.06^Aa^; 0.41±0.03^Bb^; 0.52±0.04^Bc^ | | | | | | | | | | 0.96±0.08^Aa^; 1.01±0.12^Aa^; 0.35±0.03^Bb^; 0.47±0.03^Bc^ | | | | | | | | |
| Txnrd3 | 1.00±0.00^Aa^; 0.93±0.13^Aa^; 0.55±0.04^Ab^; 0.71±0.09^Ab^ | | | | | | | 0.91±0.06^Aa^; 0.93±0.01^Aa^; 0.47±0.03^Bb^; 0.62±0.03^Ab^ | | | | | | | | | | 0.91±0.05^Aa^; 0.95±0.09^Aa^; 0.45±0.02^Bc^; 0.59±0.09^Ac^ | | | | | | | | |
| Dio1 | 1.00±0.00^Aa^; 0.98±0.01^Aa^; 0.52±0.05^Ab^; 0.80±0.08^Ac^ | | | | | | | 0.99±0.04^Aa^; 0.96±0.02^Aa^; 0.25±0.02^Bb^; 0.48±0.10^Bc^ | | | | | | | | | | 1.02±0.01^Aa^; 0.96±0.06^Aa^; 0.23±0.01^Bb^; 0.40±0.04^Bc^ | | | | | | | | |
| Dio2 | 1.00±0.00^Aa^; 0.99±0.06^Aa^; 0.45±0.01^Ab^; 0.70±0.08^Ac^ | | | | | | | 0.95±0.05^Aa^; 0.99±0.05^Aa^; 0.48±0.05^Ab^; 0.72±0.00^Ac^ | | | | | | | | | | 1.01±0.04^Aa^; 0.92±0.05^Aa^; 0.48±0.04^Ab^; 0.62±0.06^Ab^ | | | | | | | | |
| Dio3 | 1.00±0.00^Aa^; 0.95±0.06^Aa^; 0.62±0.09^Ab^; 0.78±0.02^Ab^ | | | | | | | 0.90±0.05^Aa^; 0.91±0.12^Aa^; 0.25±0.05^Bb^; 0.35±0.01^Bc^ | | | | | | | | | | 0.93±0.05^Aa^; 0.94±0.05^Aa^; 0.17±0.01^Bb^; 0.37±0.01^Bb^ | | | | | | | | |
| SelT | 1.00±0.00^Aa^; 1.09±0.11^Aa^; 0.64±0.09^Ab^; 0.78±0.06^Ac^ | | | | | | | 0.98±0.08^Aa^; 0.99±0.07^Aa^; 0.49±0.04^Ab^; 0.69±0.14^Ab^ | | | | | | | | | | 1.08±0.07^Aa^; 1.04±0.09^Aa^; 0.40±0.07^Bb^; 0.65±0.12^Ac^ | | | | | | | | |
| SelK | 1.00±0.00^Aa^; 1.03±0.08^Aa^; 0.27±0.03^Ab^; 0.56±0.09^Ac^ | | | | | | | 1.07±0.03^Aa^; 0.99±0.09^Aa^; 0.27±0.03^Ab^; 0.59±0.09^Ac^ | | | | | | | | | | 1.02±0.06^Aa^; 1.00±0.09^Aa^; 0.22±0.04^Ab^; 0.35±0.05^Bc^ | | | | | | | | |
| SelS | 1.00±0.00^Aa^; 1.05±0.05^Aa^; 0.33±0.05^Ab^; 0.61±0.09^Ac^ | | | | | | | 1.00±0.09^Aa^; 0.99±0.07^Aa^; 0.32±0.03^Ab^; 0.43±0.06^Ac^ | | | | | | | | | | 1.04±0.04^Aa^; 1.01±0.01^Aa^; 0.29±0.03^Ab^; 0.45±0.08^Ab^ | | | | | | | | |
| SelH | 1.00±0.00^Aa^; 0.95±0.06^Aa^; 0.56±0.07^Ab^; 0.60±0.06^Ab^ | | | | | | | 0.93±0.06^Aa^; 0.95±0.04^Aa^; 0.27±0.03^Bb^; 0.45±0.05^Bc^ | | | | | | | | | | 0.94±0.07^Aa^; 0.92±0.06^Aa^; 0.26±0.02^Bb^; 0.59±0.07^Bc^ | | | | | | | | |
| SelM | 1.00±0.00^Aa^; 0.92±0.03^Aa^; 0.51±0.02^Ab^; 0.81±0.05^Ac^ | | | | | | | 0.96±0.08^Aa^; 0.97±0.07^Aa^; 0.15±0.02^Bb^; 0.75±0.04^Ac^ | | | | | | | | | | 0.97±0.09^Aa^; 0.95±0.02^Aa^; 0.30±0.02^Cb^; 0.71±0.07^Ac^ | | | | | | | | |
| SelU | 1.00±0.00^Aa^; 0.95±0.06^Aa^; 0.33±0.04^Ab^; 0.78±0.04^Ac^ | | | | | | | 0.94±0.07^Aa^; 0.86±0.04^Aa^; 0.29±0.03^Ab^; 0.50±0.04^Bc^ | | | | | | | | | | 0.92±0.07^Aa^; 0.99±0.08^Aa^; 0.27±0.04^Ab^; 0.62±0.07^Bc^ | | | | | | | | |
| SelI | 1.00±0.00^Aa^; 0.97±0.07^Aa^; 0.51±0.09^Ab^; 0.61±0.03^Ab^ | | | | | | | 1.01±0.06^Aa^; 0.97±0.09^Aa^; 0.36±0.06^Bb^; 0.57±0.05^Ac^ | | | | | | | | | | 0.97±0.04^Aa^; 0.97±0.02^Aa^; 0.32±0.03^Bb^; 0.57±0.03^Ac^ | | | | | | | | |
| SelO | 1.00±0.00^Aa^; 1.01±0.05^Aa^; 0.30±0.03^Ab^; 0.76±0.10^Ac^ | | | | | | | 0.97±0.03^Aa^; 0.94±0.02^Aa^; 0.30±0.04^Ab^; 0.38±0.05^Bb^ | | | | | | | | | | 0.97±0.04^Aa^; 0.93±0.06^Aa^; 0.19±0.02^Bb^; 0.40±0.07^Bc^ | | | | | | | | |
| Selpb | 1.00±0.00^Aa^; 1.01±0.08^Aa^; 0.30±0.02^Ab^; 0.70±0.08^Ac^ | | | | | | | 1.01±0.07^Aa^; 1.00±0.03^Aa^; 0.17±0.02^Bb^; 0.62±0.14^Ac^ | | | | | | | | | | 0.91±0.05^Aa^; 0.98±0.08^Aa^; 0.14±0.02^Bb^; 0.41±0.04^Bc^ | | | | | | | | |
| Sepn1 | 1.00±0.00^Aa^; 0.95±0.11^Aa^; 0.43±0.02^Ab^; 0.68±0.05^Ac^ | | | | | | | 0.95±0.04^Aa^; 0.92±0.08^Aa^; 0.21±0.02^Bb^; 0.41±0.04^Bc^ | | | | | | | | | | 0.91±0.08^Aa^; 0.86±0.05^Aa^; 0.21±0.03^Bb^; 0.52±0.05^Bc^ | | | | | | | | |
| Sepp1 | 1.00±0.00^Aa^; 0.99±0.01^Aa^; 0.49±0.04^Ab^; 0.72±0.02^Ac^ | | | | | | | 0.93±0.06^Aa^; 0.91±0.05^Aa^; 0.15±0.02^Bb^; 0.30±0.05^Bc^ | | | | | | | | | | 0.97±0.07^Aa^; 0.98±0.02^Aa^; 0.13±0.02^Bb^; 0.26±0.04^Bc^ | | | | | | | | |
| Sepx1 | 1.00±0.00^Aa^; 0.98±0.03^Aa^; 0.43±0.02^Ab^; 0.79±0.04^Ac^ | | | | | | | 0.98±0.03^Aa^; 1.01±0.10^Aa^; 0.29±0.06^Bb^; 0.46±0.03^Bc^ | | | | | | | | | | 0.95±0.03^Aa^; 0.99±0.09^Aa^; 0.26±0.05^Bb^; 0.52±0.06^Bc^ | | | | | | | | |
| Sepw1 | 1.00±0.00^Aa^; 1.01±0.00^Aa^; 0.43±0.02^Ab^; 0.65±0.11^Ac^ | | | | | | | 0.97±0.05^Aa^; 0.96±0.02^Aa^; 0.29±0.04^Bb^; 0.46±0.03^Bc^ | | | | | | | | | | 1.05±0.04^Aa^; 0.98±0.04^Aa^; 0.26±0.03^Bb^; 0.38±0.05^Bc^ | | | | | | | | |
| Sep15 | 1.00±0.00^Aa^; 0.97±0.04^Aa^; 0.47±0.03^Ab^; 0.69±0.02^Ac^ | | | | | | | 0.98±0.05^Aa^; 1.04±0.05^Aa^; 0.19±0.01^Bb^; 0.50±0.02^Ac^ | | | | | | | | | | 1.00±0.08^Aa^; 1.06±0.03^Aa^; 0.16±0.02^Bb^; 0.52±0.03^Ac^ | | | | | | | | |
| SPS2 | 1.00±0.00^Aa^; 0.99±0.04^Aa^; 0.45±0.06^Ab^; 0.69±0.08^Ac^ | | | | | | | 0.99±0.07^Aa^; 0.96±0.05^Aa^; 0.44±0.02^Ab^; 0.65±0.05^Ac^ | | | | | | | | | | 0.93±0.07^Aa^; 0.98±0.01^Aa^; 0.41±0.03^Ab^; 0.59±0.04^Ac^ | | | | | | | | |
