# Supplementary material for: Selenium against lead-induced apoptosis in chicken nervous tissues via mitochondrial pathway
Source: Oncotarget. 2017 Nov 20;8(64):108130–45. doi: 10.18632/oncotarget.22553 (PMC5746131; doi:10.18632/oncotarget.22553)
Supplement: Supplementary file 3 [file oncotarget-08-108130-s003.docx]

**Supplementary Table 2: The relative mRNA expression of twenty-five selenoproteins in the chicken embryonic neurocytes**

| **Seleno-**  **proteins** |  | **12 hours** | | | |  |  | **24 hours** | | | |  |  | **36 hours** | | | |  |  | **48 hours** | | | |  |
| --- | --- | --- | --- | --- | --- | --- | --- | --- | --- | --- | --- | --- | --- | --- | --- | --- | --- | --- | --- | --- | --- | --- | --- | --- |
|  |  | **Control** | **Se** | **Pb** | **Se/Pb** |  |  | **Control** | **Se** | **Pb** | **Se/Pb** |  |  | **Control** | **Se** | **Pb** | **Se/Pb** |  |  | **Control** | **Se** | **Pb** | **Se/Pb** |  |
| Gpx1 | 1.00±0.00^Aa^; 1.06±0.09^Aa^; 0.43±0.01^Ab^; 0.54±0.02^Ab^ | | | | | | 1.06±0.09^Aa^; 1.02±0.07^Aa^; 0.43±0.02^Ab^; 0.53±0.05^Ab^ | | | | | | 0.95±0.06^Aa^; 0.94±0.03^Aa^; 0.44±0.02^Ab^; 0.52±0.09^Ab^ | | | | | | 0.96±0.09^Aa^; 0.95±0.03^Aa^; 0.45±0.07^Ab^; 0.46±0.04^Ab^ | | | | | |
| Gpx2 | 1.00±0.00^Aa^; 1.02±0.11^Aa^; 0.42±0.22^Ab^; 0.44±0.23^Ab^ | | | | | | 1.08±0.05^Aa^; 1.08±0.01^Aa^; 0.29±0.04^Ab^; 0.51±0.05^Ac^ | | | | | | 1.05±0.08^Aa^; 1.01±0.15^Aa^; 0.38±0.02^Ab^; 0.47±0.08^Ab^ | | | | | | 1.04±0.12^Aa^; 1.00±0.09^Aa^; 0.35±0.03^Ab^; 0.43±0.01^Ab^ | | | | | |
| Gpx3 | 1.00±0.00^Aa^; 0.98±0.07^Aa^; 0.60±0.04^Ab^; 0.68±0.04^Ab^ | | | | | | 0.98±0.07^Aa^; 0.96±0.06^Aa^; 0.44±0.13^Ab^; 0.74±0.01^Ac^ | | | | | | 1.02±0.11^Aa^; 0.98±0.05^Aa^; 0.43±0.04^Ab^; 0.71±0.06^Ac^ | | | | | | 1.01±0.01^Aa^; 0.99±0.08^Aa^; 0.42±0.05^Ab^; 0.64±0.04^Ac^ | | | | | |
| Gpx4 | 1.00±0.00^Aa^; 1.02±0.04^Aa^; 0.35±0.05^Ab^; 0.49±0.05^Ac^ | | | | | | 1.06±0.03^Aa^; 1.02±0.06^Aa^; 0.42±0.03^Ab^; 0.47±0.02^Ab^ | | | | | | 1.05±0.06^Aa^; 1.02±0.05^Aa^; 0.39±0.05^Ab^; 0.56±0.04^Ab^ | | | | | | 1.05±0.03^Aa^; 1.08±0.01^Aa^; 0.43±0.04^Ab^; 0.55±0.05^Ac^ | | | | | |
| Txnrd1 | 1.00±0.00^Aa^; 1.10±0.13^Aa^; 0.50±0.03^Ab^; 0.46±0.02^Ab^ | | | | | | 1.03±0.04^Aa^; 1.04±0.02^Aa^; 0.43±0.03^Bb^; 0.50±0.01^Ab^ | | | | | | 1.02±0.05^Aa^; 1.08±0.01^Aa^; 0.33±0.02^Cb^; 0.49±0.04^Ac^ | | | | | | 1.07±0.03^Aa^; 0.99±0.02^Aa^; 0.33±0.02^Cb^; 0.43±0.05^Ac^ | | | | | |
| Txnrd2 | 1.00±0.00^Aa^; 1.04±0.01^Aa^; 0.38±0.06^Ab^; 0.65±0.05^Ac^ | | | | | | 1.04±0.06^Aa^; 0.97±0.05^Aa^; 0.43±0.05^Ab^; 0.62±0.08^Ac^ | | | | | | 1.04±0.04^Aa^; 0.98±0.07^Aa^; 0.46±0.14^Ab^; 0.61±0.06^Ab^ | | | | | | 1.04±0.03^Aa^; 1.08±0.09^Aa^; 0.51±0.05^Ab^; 0.67±0.06^Ac^ | | | | | |
| Txnrd3 | 1.00±0.00^Aa^; 1.00±0.06^Aa^; 0.27±0.00^Ab^; 0.42±0.03^Ac^ | | | | | | 1.04±0.04^Aa^; 1.00±0.04^Aa^; 0.28±0.04^Ab^; 0.42±0.06^Ac^ | | | | | | 0.98±0.05^Aa^; 0.96±0.04^Aa^; 0.34±0.03^Ab^; 0.47±0.02^Ab^ | | | | | | 1.00±0.02^Aa^; 1.01±0.05^Aa^; 0.40±0.09^Ab^; 0.44±0.01^Ab^ | | | | | |
| Dio1 | 1.00±0.00^Aa^; 1.05±0.03^Aa^; 0.48±0.00^Ab^; 0.54±0.03^Ab^ | | | | | | 1.03±0.04^Aa^; 1.05±0.04^Aa^; 0.45±0.01^Ab^; 0.44±0.04^Bb^ | | | | | | 1.02±0.02^Aa^; 1.01±0.04^Aa^; 0.42±0.01^Bb^; 0.43±0.03^Bb^ | | | | | | 1.01±0.04^Aa^; 0.96±0.03^Aa^; 0.36±0.02^Cb^; 0.37±0.03^Bb^ | | | | | |
| Dio2 | 1.03±0.04^Aa^; 1.09±0.01^Aa^; 0.52±0.02^Ab^; 0.54±0.02^Ab^ | | | | | | 1.03±0.04^Aa^; 1.03±0.02^Aa^; 0.48±0.02^Ab^; 0.52±0.01^Ab^ | | | | | | 1.05±0.05^Aa^; 1.04±0.04^Aa^; 0.42±0.02^Bb^; 0.51±0.04^Ab^ | | | | | | 1.01±0.09^Aa^; 1.02±0.03^Aa^; 0.38±0.03^Bb^; 0.53±0.04^Ac^ | | | | | |
| Dio3 | 1.00±0.00^Aa^; 1.02±0.03^Aa^; 0.43±0.04^Ab^; 0.70±0.06^Ac^ | | | | | | 1.05±0.05^Aa^; 1.01±0.03^Aa^; 0.46±0.02^Ab^; 0.67±0.04^Ac^ | | | | | | 1.06±0.03^Aa^; 1.03±0.03^Aa^; 0.40±0.02^Bb^; 0.54±0.04^Ac^ | | | | | | 1.06±0.04^Aa^; 1.00±0.03^Aa^; 0.42±0.02^Cb^; 0.38±0.03^Ac^ | | | | | |
| SelT | 1.00±0.00^Aa^; 0.99±0.01^Aa^; 0.42±0.01^Ab^; 0.61±0.01^Ac^ | | | | | | 1.05±0.01^Aa^; 1.04±0.03^Aa^; 0.36±0.03^Ab^; 0.58±0.00^Ac^ | | | | | | 0.99±0.07^Aa^; 1.04±0.02^Aa^; 0.36±0.04^Ab^; 0.53±0.05^Ac^ | | | | | | 1.04±0.02^Aa^; 1.00±0.02^Aa^; 0.36±0.04^Ab^; 0.51±0.06^Ac^ | | | | | |
| SelK | 1.00±0.00^Aa^; 1.04±0.02^Aa^; 0.49±0.04^Ab^; 0.81±0.01^Ac^ | | | | | | 1.04±0.05^Aa^; 1.04±0.04^Aa^; 0.52±0.01^Ab^; 0.77±0.01^Ac^ | | | | | | 1.08±0.03^Aa^; 1.04±0.03^Aa^; 0.35±0.03^Bb^; 0.45±0.02^Bc^ | | | | | | 0.99±0.06^Aa^; 0.98±0.07^Aa^; 0.26±0.03^Cb^; 0.43±0.08^Bc^ | | | | | |
| SelS | 1.00±0.00^Aa^; 0.98±0.06^Aa^; 0.58±0.03^Ab^; 0.78±0.02^Ac^ | | | | | | 1.02±0.07^Aa^; 1.04±0.05^Aa^; 0.55±0.02^Ab^; 0.57±0.06^Bb^ | | | | | | 1.01±0.04^Aa^; 1.05±0.02^Aa^; 0.42±0.02^Bb^; 0.53±0.07^Bb^ | | | | | | 0.97±0.07^Aa^; 0.96±0.07^Aa^; 0.40±0.05^Bb^; 0.50±0.07^Bb^ | | | | | |
| SelH | 1.00±0.00^Aa^; 1.04±0.05^Aa^; 0.51±0.02^Ab^; 0.66±0.03^Ac^ | | | | | | 1.05±0.03^Aa^; 1.06±0.02^Aa^; 0.49±0.10^Ab^; 0.58±0.03^Ab^ | | | | | | 1.02±0.06^Aa^; 0.98±0.04^Aa^; 0.49±0.07^Ab^; 0.48±0.01^Bb^ | | | | | | 1.00±0.06^Aa^; 0.96±0.05^Aa^; 0.42±0.02^Ab^; 0.45±0.01^Bb^ | | | | | |
| SelM | 1.00±0.00^Aa^; 1.03±0.01^Aa^; 0.71±0.02^Ab^; 0.80±0.02^Ac^ | | | | | | 1.02±0.02^Aa^; 1.00±0.05^Aa^; 0.68±0.03^Ab^; 0.78±0.06^Ab^ | | | | | | 1.02±0.02^Aa^; 1.03±0.06^Aa^; 0.52±0.02^Bb^; 0.63±0.04^Bc^ | | | | | | 1.05±0.02^Aa^; 1.03±0.04^Aa^; 0.52±0.05^Bb^; 0.61±0.03^Bb^ | | | | | |
| SelU | 1.00±0.00^Aa^; 0.98±0.01^Aa^; 0.58±0.02^Ab^; 0.58±0.04^Ac^ | | | | | | 1.03±0.08^Aa^; 1.04±0.01^Aa^; 0.52±0.03^Bb^; 0.57±0.01^Ab^ | | | | | | 0.99±0.09^Aa^; 1.04±0.04^Aa^; 0.42±0.01^Cb^; 0.55±0.03^Ab^ | | | | | | 1.04±0.01^Aa^; 1.03±0.05^Aa^; 0.34±0.03^Db^; 0.54±0.03^Ab^ | | | | | |
| SelI | 1.00±0.00^Aa^; 0.96±0.08^Aa^; 0.36±0.02^Ab^; 0.58±0.03^Ac^ | | | | | | 1.04±0.01^Aa^; 1.02±0.07^Aa^; 0.35±0.03^Ab^; 0.50±0.07^Bc^ | | | | | | 1.01±0.04^Aa^; 1.03±0.08^Aa^; 0.38±0.01^Ab^; 0.39±0.04^Bb^ | | | | | | 1.03±0.04^Aa^; 1.05±0.05^Aa^; 0.36±0.02^Ab^; 0.46±0.02^Bc^ | | | | | |
| SelO | 1.00±0.00^Aa^; 1.04±0.03^Aa^; 0.50±0.04^Ab^; 0.63±0.08^Ab^ | | | | | | 1.03±0.01^Aa^; 1.04±0.03^Aa^; 0.52±0.07^Ab^; 0.53±0.02^Ab^ | | | | | | 0.99±0.06^Aa^; 0.97±0.07^Aa^; 0.45±0.07^Ab^; 0.48±0.13^Ab^ | | | | | | 0.99±0.08^Aa^; 0.98±0.06^Aa^; 0.44±0.15^Ab^; 0.48±0.02^Ab^ | | | | | |
| Selpb | 1.00±0.00^Aa^; 0.96±0.06^Aa^; 0.40±0.05^Ab^; 0.77±0.03^Ac^ | | | | | | 1.03±0.03^Aa^; 1.05±0.03^Aa^; 0.32±0.05^Ab^; 0.55±0.04^Bc^ | | | | | | 1.05±0.04^Aa^; 1.03±0.03^Aa^; 0.35±0.05^Ab^; 0.55±0.03^Bc^ | | | | | | 1.01±0.05^Aa^; 1.04±0.06^Aa^; 0.37±0.04^Ab^; 0.53±0.03^Bc^ | | | | | |
| Sepn1 | 1.00±0.00^Aa^; 1.02±0.02^Aa^; 0.39±0.05^Ab^; 0.56±0.03^Ac^ | | | | | | 1.01±0.08^Aa^; 0.99±0.03^Aa^; 0.39±0.01^Ab^; 0.54±0.02^Ab^ | | | | | | 1.02±0.04^Aa^; 1.05±0.09^Aa^; 0.37±0.04^Ab^; 0.46±0.01^Bb^ | | | | | | 1.02±0.02^Aa^; 1.00±0.06^Aa^; 0.32±0.02^Ab^; 0.38±0.04^Bb^ | | | | | |
| Sepp1 | 1.00±0.00^Aa^; 0.94±0.09^Aa^; 0.52±0.02^Ab^; 0.70±0.04^Ac^ | | | | | | 0.95±0.06^Aa^; 1.00±0.08^Aa^; 0.44±0.03^Bb^; 0.56±0.06^Bb^ | | | | | | 1.01±0.05^Aa^; 0.99±0.05^Aa^; 0.42±0.02^Bb^; 0.55±0.03^Bc^ | | | | | | 1.04±0.06^Aa^; 0.97±0.03^Aa^; 0.46±0.03^Bb^; 0.54±0.05^Bb^ | | | | | |
| Sepx1 | 1.00±0.00^Aa^; 1.01±0.03^Aa^; 0.43±0.02^Ab^; 0.68±0.01^Ac^ | | | | | | 1.05±0.03^Aa^; 1.02±0.05^Aa^; 0.42±0.07^Ab;^ 0.50±0.11^Ab^ | | | | | | 1.04±0.03^Aa^; 1.05±0.01^Aa^; 0.38±0.03^Ab^; 0.59±0.06^Ac^ | | | | | | 1.04±0.02^Aa^; 1.03±0.06^Aa^; 0.32±0.03^Ab^; 0.57±0.05^Ac^ | | | | | |
| Sepw1 | 1.00±0.00^Aa^; 1.05±0.02^Aa^; 0.53±0.01^Ab^; 0.86±0.04^Ac^ | | | | | | 0.98±0.03^Aa^; 0.95±0.14^Aa^; 0.57±0.01^Ab^; 0.63±0.02^Bb^ | | | | | | 1.07±0.03^Aa^; 1.01±0.05^Aa^; 0.44±0.03^Ab^; 0.63±0.02^Bc^ | | | | | | 1.02±0.08^Aa^; 0.99±0.02^Aa^; 0.47±0.09^Ab^; 0.56±0.05^Bb^ | | | | | |
| Sep15 | 1.00±0.00^Aa^; 1.01±0.02^Aa^; 0.65±0.01^Ab^; 0.79±0.02^Ac^ | | | | | | 0.97±0.05^Aa^; 0.99±0.05^Aa^; 0.54±0.02^Ab^; 0.57±0.00^Ac^ | | | | | | 1.05±0.02^Aa^; 1.02±0.05^Aa^; 0.45±0.01^Ab^; 0.57±0.01^Ac^ | | | | | | 1.03±0.05^Aa^; 1.01±0.05^Aa^; 0.42±0.06^Ab^; 0.56±0.05^Ac^ | | | | | |
| SPS2 | 1.00±0.00^Aa^; 1.03±0.03^Aa^; 0.43±0.01^Ab^; 0.71±0.01^Ac^ | | | | | | 1.04±0.04^Aa^; 1.04±0.02^Aa^; 0.49±0.06^Ab^; 0.71±0.06^Bb^ | | | | | | 1.01±0.02^Aa^; 1.03±0.02^Aa^; 0.45±0.05^Ab^; 0.69±0.07^Bc^ | | | | | | 1.00±0.02^Aa^; 1.05±0.03^Aa^; 0.44±0.01^Ab^; 0.64±0.03^Bb^ | | | | | |
